# Helping Children Cope with Loss: Legacy Interventions for the Grieving Classroom

**DOI:** 10.5334/cie.45

**Published:** 2022-09-05

**Authors:** Annie Lawrence, Maile Jones, Jessika Boles

**Affiliations:** 1 Monroe Carell Jr. Children’s Hospital at Vanderbilt, US; 2 Vanderbilt University, US

**Keywords:** Death, coping, legacy, teacher, school, children

## Abstract

Early experiences of death and loss have a significant impact on children’s coping and development across the lifespan, whether the deceased was a family member, friend, or even classmate. Given the sense of community and continuity that children often garner in schools, teachers are uniquely positioned to tailor and facilitate grief supports to meet the developmental and coping needs of their students in response to loss, especially in the case of a classmate’s death. Legacy building interventions, though healthcare-derived, have internationally been applied to promote self-expression and meaning making for grieving children and families; the underlying theory and practice of such interventions may render them useful even in classroom environments. Combining art-based activities with storytelling and reflection, legacy building activities may promote awareness and understanding of death in children and adolescents while encouraging adaptive grief responses. This paper describes how teachers may apply legacy building interventions to support students experiencing the death of a classmate. Two sample curricular plans are provided, one targeted towards elementary school children, the other towards middle and high school students. Although these resources appear ready for immediate implementation, it is essential that teachers adapt them – based on their unique classroom, school system, and sociocultural context – to best meet the needs of grieving students.

Early experiences of bereavement or loss can have significant and variable impacts on children’s coping and development. Research has shown that children’s experiences with the death of a loved one can adversely affect their developmental trajectory, thereby impacting academic achievements (both cognitive and social-emotional) in the classroom (Levkovich & Elyoseph, 2021). Emotional distress symptoms such as sadness, shock, and confusion are also rampant among children actively grieving the death of a friend or family member (Case et al., 2020; Mak, 2013; Tillman & Prozak, 2018). Additional empirical evidence suggests that bereaved children are at elevated risk of poor physical and psychological outcomes throughout the grief process (Levkovich & Elyoseph, 2021; Mak, 2013), the length of which often varies based on the child’s individual grief needs and the supports perceived to be available (Compas et al., 2012).

While many children have both the internal skills and external supports needed to affect adaptive grief efforts, some children, especially those with limited social resources, remain at high risk of adverse and enduring psychological and physiological responses incurred by bereavement (Steffen et al., 2020). Prolonged or poorly managed grief in childhood can increase the child’s likelihood of developing depression, anxiety, post-traumatic stress disorder, somatic complaints, and behavioral problems long after the loss has occurred (Levkovich & Elyoseph, 2021; Mak, 2013). Thus, the experience of grief has visible effects on both current functioning and future psychosocial wellbeing throughout the lifespan.

To adequately understand children’s grief experiences, and bolster their coping with death, a developmental and dynamic perspective is imperative; through this lens, the child is an active, changing agent in a continuous and dynamic relationship with their environment (Compas et al., 2012). Typically, the critical factor in defining stress and one’s ability to cope with loss lays largely in how the person perceives the relevance of the situation to their own well-being, as observed in both children and adults (Compas et al., 2012; Hitchcock et al., 2015). A developmental framework is also needed to anticipate how conceptions of death as final, irreversible, and universal develop from childhood into adolescence. Since death is an ambiguous concept with concrete manifestations and effects, complete understanding takes years of cognitive and emotional growth and social exposure. Additionally, a culturally responsive orientation is crucial, as with many health-related and existential concepts such as death, there is profound cultural variation in if, how, and when these elements should be discussed, addressed, or treated.

While developmental theory offers a general context for how children understand death, there can still be great variation in children’s understanding of death even within a single developmental stage. For example, a younger child who has experienced illness or death intimately through lived experience will likely have a deeper understanding of these concepts than an older child with no personal experience (Bluebond-Langner et al., 2010). Also, those with more exposure to illness, hospitalization, and medical treatment, either themselves or within their family context, may similarly have more in-depth knowledge than those who are naïve in this regard. Thus, although a developmental frame of reference is useful in assessing children’s understanding of death, it is important to meet children where they are in their experiences rather than adhering to what may be somewhat arbitrary stage models of cognitive or psychosocial development.

Acknowledging the immediate and long-term developmental impacts of a death event, its universality and finality, and its pursuant grief process, psychosocial interventions are ideal for promoting emotional wellbeing and supporting effective coping in children and adolescents who have experienced the loss of a loved one. More specifically, cultivating adaptive and effective coping skills is the primary mechanism for reducing emotional, behavioral, and academic distress among children experiencing loss. When children are given the tools to accurately appraise and overcome stressful situations and experiences, such as the death of a family member, friend, or even classmate, they are more likely to experience emotional healing that builds resilience across all domains of life (Boles et al., 2020a; Compas et al., 2012).

One component of the appraisal and coping process is children’s knowledge of the death event and the meaning of death. In Western cultures, specifically, it is suggested that children need access to honest, accurate, and developmentally appropriate medical information to build the resilience needed to cope with grief (Eklund et al., 2021; Kronaizl, 2019; Martinčeková et al., 2018; Rapa et al., 2020). However, such transparency and direct communication is not valued or encouraged in all sociocultural contexts. Therefore, it is important that information is both (a) culturally and individually tailored and (b) paired with culturally responsive social supports to best help children and adolescents cope with and make sense of death.

The death of a friend or family member is a significant developmental, educational, and psychological experience for children and adolescents that – when well attended to – can promote adaptive coping and resilience in stressful situations across the lifespan. Therefore, the purpose of this paper is to describe how teachers can use a particular type of psychosocial intervention called “legacy building” to help their students understand, express their feelings about, and make meaning of a personal loss such as the death of a classmate. After describing the development of, evidence regarding, and current practices for legacy interventions in healthcare settings, we introduce two specific curricular plans that educators can adapt for use in their elementary, middle, and high school classrooms.

## Legacy: Theory and Practice

“Legacy building” is a strategy utilized in healthcare settings to enhance coping and help patients and families create a legacy, or make meaning out of experiences (Boles, 2014; Boles et al., 2021; Foster et al., 2009). While at times also referred to as “legacy-making” (Foster et al., 2012) or “memory-making” (Steffen et al., 2020), legacy building interventions have historically been grounded in the concept of legacy as doing or saying something to be remembered (Allen et al., 2008), and have been adapted for use across North America, Europe, Asia, and Australia for adult and pediatric patients and families (Boles & Jones, 2021). Often provided by Certified Child Life Specialist and creative arts therapists or allied healthcare professionals, legacy building interventions are interactional and collaborative techniques for promoting connection, self-expression, and meaning making while creating lasting positive memories (Boles et al., 2021; Cahalan et al., 2022). Though they can take many forms, hospitals most commonly facilitate legacy building interventions at or near end of life (Allen et al., 2008; Foster et al., 2012).

Legacy-oriented interventions (inclusive of legacy building, legacy-making, and other related nomenclature) have been explored in both adult and pediatric healthcare settings in four continents. In adult settings, legacy interventions have primarily been conducted during the end-of-life period to promote adaptive coping and anticipatory grief for both patients and family members. Research evaluating Chochinov’s (2005) Dignity Therapy demonstrated decreased physical suffering and depressive symptoms, as well as increased will to live, among elderly patients who participated in the legacy intervention (Montross et al., 2011). Additionally, Allen et al.’s (2008) Life Review intervention decreased caregiver stress and increased social engagement of participants. Both interventions used a family-centered approach that prompted intentional reflection on the patient’s medical experiences and collaborative meaning making – elements that are often present in pediatric legacy interventions.

In pediatric hospitals and healthcare settings, legacy building interventions are often presented as the standardized creation of keepsakes such as plaster hand molds and handprints intended to provide bereaved family members with a tangible way to remember the dying child (Foster et al., 2012). Children and families can intentionally and meaningfully create and document legacy through journals, memory boxes, artwork, scrapbooks, and letters (Akard et al., 2021; Schaefer, 2019; Sisk 2012). Creating legacy documents in this way aim to help bereaved friends and family members connect, remember, and feel a sense of control amidst the stress of death and loss of a loved one (Allen et al., 2008; Foster et al., 2012).

Legacy as a concept and clinical practice can also be developed and created serendipitously, and represented by intangible items such as character traits, beliefs, actions, stories, and values (Boles et al., 2020b, 2021; Jones et al., 2022). A recent reframing of legacy has emerged to represent legacy as “avenues of connection, education, inspiration, or transformation” (Boles & Jones, 2021, p. 19). Consistent with the emerging literature representing legacy as a continual process of meaning making, the idea of legacy living – or the use of legacy-oriented interventions throughout the illness and treatment trajectory – has been shown to improve quality of life, increase communication, decrease emotional suffering, and reduce psychological distress (Cahalan et al., 2022).

Though a legacy itself and legacy-oriented interventions can take a variety of forms, the value comes not from the product it creates but rather the process, which promotes connection, collaboration, and control (Boles, 2014). In this sense, legacy interventions are intended to promote adaptive grief processes, facilitate supportive conversations, foster emotional expression, and cultivate continuing bonds between the living and the deceased (Boles et al., 2021; Jones et al., 2022). Whether intentional or serendipitous, efforts to build memories and make meaning out of difficult experiences can be an important gateway for coping. When practiced through a goodness-of-fit model, which requires intentional assessment of legacy perceptions, goals, and preferences for the child and family, legacy building interventions are optimally therapeutic and effective.

## Bringing Legacy Interventions to the Classroom

The school setting is one of the most crucial contexts for development in school-aged children and adolescents. Not only does the school environment occupy a significant portion of the child’s time, but it propels a child’s cognitive, psychosocial, emotional, and physical development in a myriad of ways (Boonen & Petry, 2011); such comprehensive gains are achieved through both targeted educational interventions and the intentional and thoughtful inclusion of “real-world” lessons and social connections for learning facilitated by the classroom teacher.

Throughout children’s years of formal schooling, many will encounter traumatic experiences, such as serious illness or death, familial conflict and discord, community violence, or natural disasters. While these topics may not be conventionally discussed in the classroom, educators – as well trusted adults – are uniquely positioned to hold safe space to discuss these issues within the walls of the classroom. The importance of providing open and honest dialogue around these issues may be especially imperative when the entirety of the class or school is impacted, as is the case with the death of a classmate.

When a child dies, the loss reverberates in the various systems and communities in which the child was engaged. The deceased child’s classmates, and larger school community, are acutely impacted when there is such a monumental change and absence (Case et al., 2020). Because the school setting provides children with a sense of normalcy and familiarity, disruption to this equilibrious system can feel especially disorienting (Levkovich & Elyoseph, 2021; Weiner et al., 2018). At the same time, the growing acknowledgment of schools as compassionate communities (Quinn et al., 2021) suggests that the time has come to consider school-based interventions for grief support after a student’s death.

For children to adaptively cope with the loss of a classmate, it is important that they are exposed to salient adult models that exhibit these grief behaviors and coping tactics. With targeted and developmentally appropriate psychoeducation, intentional provision of therapeutic activities such as bibliotherapy and legacy building, educators can equip students with the tools they need to process these complex emotions. Furthermore, by fostering open dialogue with students, educators can acknowledge, validate, and normalize the wide range of emotions that children either are already or experiencing or may come to feel at some point in the future. In the same way that each student will have had a unique relationship with the deceased child, so too will their grief response be distinct; the grieving process is also informed by a variety of factors, including the depth of the relationship, the child’s developmental level, and their previous experiences with death and grief (Mak, 2013). Therefore, an individualized approach should be utilized to meet the developmental and emotional needs of all students.

Although the literature describes the psychosocial benefits associated with open and honest conversations around death in many sociocultural contexts, many teachers report feeling unprepared for and ill equipped to facilitate these pivotal conversations (DeMuth et al., 2020). When the topic of death is not acknowledged and discussed, children may feel alone and isolated as they navigate the complex emotional responses of grief; in fact, children are likely to infer that just as the concept of death is not open for discussion so too are their feelings and thoughts about the death event. Additionally, if their questions and concerns surrounding death are left unheard and unanswered, children may develop misconceptions that exacerbate the risk of negative psychological outcomes (Benner & Marlow, 1991; Clay et al., 2004).

Noting these potential difficulties, a multidisciplinary approach to children’s grief is essential not only in healthcare setting, but also in classroom environments. Ideally, collaboration between (a) hospital-based support staff such as certified child life specialists (with expertise in child development and coping), (b) hospital-based mental health practitioners such as school psychologists or social workers, and (c) educators with whom children have a strong familiarity, rapport, and trust, is the first step in designing activities and interventions for grieving students. In the context of such collaboration, and in efforts to help educators feel more comfortable facilitating educational and therapeutic conversations for students after a classmate’s death, we have developed two evidence informed legacy building lesson plans as a flexible and customizable scaffold for grief support in the classroom setting.

## Legacy Building Interventions for the Classroom

Multidisciplinary research evidence and systematic observations suggest that the loss of a classmate, as a close-proximity peer, initiates a grief response that some children are better equipped to manage – namely children who perceive high levels of social support and demonstrate an age-appropriate understanding of what death means. However, rarely is a singular classroom homogeneous enough to account for the variations in knowledge and coping skills children present. Therefore, just as individualized instructional strategies are necessary in a truly inclusive classroom, so too are socially and emotionally driven interventions for the grieving children who might occupy the classroom. Although legacy building interventions have primarily been studied in healthcare settings to date, their philosophical underpinnings, resilience orientation, collaborative nature, and flexible delivery can help classroom teachers – in tandem with other community and school-based support professionals – to meet the educational and coping needs of their students after the death of a classmate.

Noting the significant developmental differences between typically developing school-aged children and adolescents, we present two legacy building curriculum plans (Appendices A and B) – not as rigid, step-by-step guides but rather as a sort of scaffolding that can be adapted based on the teacher’s in-depth knowledge of their student population and classroom culture. Given the emotional intensity of the grief process, implementation of these activities requires not only attention to the active components of the exercise and interaction, but also attunement to the classroom environment and students’ cues throughout. Therefore, we recommend dedicating at least 45 minutes of classroom time for the activity, preferably at a time when the students seem most apt to engage in reflective, creative, or expressive exercises – the timing of which may differ from one classroom to the next. Additionally, it is important to ensure that this dedicated time is uninterrupted, takes place in a physically and emotionally safe classroom space, and is accompanied by an opportunity to “cool down” or take a short break before transitioning out of the legacy building intervention and back into standard curriculum.

## Considerations for Implementation

Given that death not only affects friends and classmates but also the macrocosms and systems of the child’s life, it is imperative to coordinate legacy building interventions from an organizational perspective – planning and providing different levels and types of support within and beyond the school. School administration and educators should communicate with students’ parents prior to facilitating the activity to obtain consent for their child to participate; such activities should not be mandatory and should not be included in curricular evaluations of the child’s performance. It is recommended that educators send intentional communication to students’ parents to explain the purpose of facilitating legacy interventions, the planned day and time for the child’s participation, and the importance of continuing the conversation at home.

Once teachers have received this information, it is equally important for them to offer parents the choice for their child to abstain from the legacy intervention and to honor this decision regardless of the parents’ rationale for doing so. In some cases, it may be helpful to offer parents the opportunity to individually facilitate such an experience with their child at home; thus, teachers can anticipate this potential request and make materials available for parents to use when and where they are most comfortable.

Before planning and implementing legacy building curriculum or lesson plans, educators should assess students’ knowledge and understanding of death and any pertinent details surrounding the death of their classmate. In the event that some children and parents are unaware of the child’s death, the school may want to formally communicate with families that a child in the classroom has died, triggering support mechanisms available at home while also being mindful of the deceased child and family’s privacy. Information should be provided about supports available within the school and the larger community for children and their parents, as well as preparation for the teacher’s delivery of a legacy building lesson plan. Teachers should then prepare children for what they will experience during the legacy building activity and ensure that adequate space, time, and materials are provided. Additional follow-up information about available school- and community-based grief supports should be provided to families regularly as part of the child’s ongoing education and care.

Many teachers report not feeling prepared or comfortable discussing death, or do not feel emotionally able to tackle this topic with their students (Case et al., 2020). Thus, it is important for administrators and support staff to help teachers first recognize and adapt to their own grief as they plan to provide similar support catered to the needs of their students. Additionally, teachers could benefit from staff education to help them identify signs and symptoms of normative and traumatic grief in their student population, allowing them to monitor their students’ needs more effectively throughout the school day. Such education should also include concrete strategies to help teachers foster a safe environment for disclosure and self-expression, while modeling adaptive grief responses.

With younger children, adults may feel unsure of how to broach such an abstract and existential topic as death in a clear and supportive way. Especially for young children, books can provide a nonthreatening medium for engaging in challenging topics; they have proven to be a therapeutic tool for education and coping support with children who are processing the death of a loved one. Books can help children process their own emotional, social, and spiritual concerns in a child-friendly and appealing format. Also, books provide a safe space for adult support persons, like teachers, to find the appropriate language and frame of reference to engage the child accordingly (Arruda-Colli et al., 2017). As children personally identify with characters in a book, they begin to view their own experiences as normal and important, thus opening the door for ongoing discussion.

Collaboration between the child’s family, healthcare team, and educational staff is necessary for effective assessing of needs and providing grief supports for students. Creating time and space to provide parent education is essential given that they often report feeling uncertain about how to comfort their children during a loss, as well as a sense of powerless and heightened emotional distress as a result (Cahalan et al., 2022). Thus, educators should provide developmentally appropriate take-home resources for parents to equip them with the information, tools, and skills needed to support their child’s ongoing grief.

## Evaluation

Given the individualized nature of grief and its associated responses, standardized evaluation techniques and tools are difficult to apply in this context. Attention to students’ emotional and behavior cues is essential; it is important for the teacher to be able to distinguish between normative distress and more acute distress that would require immediate and more intensive intervention. For instance, if a child becomes so overwhelmed with emotion that they cannot participate in the activity or express their needs, or if their distress is difficult to calm or console after the conclusion of the activity, this is a sign that more emotional support is needed. Thus, it is important for the school counselor and perhaps nearby classroom teachers to be aware of the lesson plan and be available to provide additional support should such a need arise.

To evaluate students’ experience of the lesson plan, teachers can assign a journal entry or short reflective paper in which students describe their perceptions of the activities and understanding of the lesson content. It is also helpful to send a note home to parents about how to support grieving children and adolescents, potentially with information about how to reach the teacher and school counselor for more support or assistance. Finally, students should be encouraged to share their artwork and/or their journal entry or reflective paper assignment with their parents or caregivers to promote continued self-expression and reflective dialogue within the family system.

## Conclusion

Continuity between hospitals and classrooms is crucial for children’s development, especially in the event of death, as children carry grief and trauma with them through all the environments they occupy and experience. The school setting plays a major role in a child’s life, acting as an anchor point to facilitate adjustment (Levkovich & Elyoseph, 2021). In the unique case of a classmate’s death, it is essential that students have access to honest, accurate, and developmentally appropriate medical information as appropriate for their cultural context, as well as culturally responsive community-based psychological supports to build the resilience needed to cope with grief.

Additionally, it is imperative to provide children with a safe environment that promotes a sense of community, affiliation, and normalization to promote adaptive healing and facilitate meaning making (Steffen et al., 2020). Educators – as well trusted adults – are well positioned to hold safe space to discuss these issues within the walls of the classroom. Given the sense of community and continuity that children experience in classroom settings, teachers are uniquely positioned to provide and adapt legacy interventions to the developmental and coping needs of their students.

Although the specific curricular plans provided here have not been empirically evaluated, recent research and developing theory suggest that legacy building interventions provide children with a valuable opportunity to reflect, express their feelings, and access resilience by making meaning of their loss experience (Boles & Jones, 2021; Cahalan et al., 2022). Combining art-based activities with storytelling and reflection, legacy building activities can promote awareness and understanding of death while encouraging adaptive grief responses and grief-related interactions in children and adolescents. Empirical evidence demonstrates that legacy building interventions have significant and cross-cultural effects on adult and pediatric patient experiences with, and family perceptions of and experiences with, the end of life – signifying a similar utility when provided after a sudden loss, such as in the death of a classmate (Boles et al., 2021; Jones et al., 2022). Research is currently underway to examine family use of legacy building interventions in healthcare settings, with plans to extend into specific patient and family subpopulations and community contexts like the school environment.

## Additional Files

The additional files for this article can be found as follows:

10.5334/cie.45.s1 Appendix A. Elementary school legacy building sample lesson plan.

10.5334/cie.45.s2 Appendix B. Middle school and high school legacy building sample lesson plan.
