# Supplementary material for: Helping Children Cope with Loss: Legacy Interventions for the Grieving Classroom
Source: Contin Educ. 2022 Sep 5;3(1):92–100. doi: 10.5334/cie.45 (PMC11104402; doi:10.5334/cie.45)
Supplement: Appendix A. — Elementary school legacy building sample lesson plan. [file cie-3-1-45-s1.pdf]

Appendix A
Elementary School Legacy Building Sample Lesson Plan

Lesson Plan on Loss: *Ida, Always*
Subject: English Language Arts
Grades: 2-5
Lesson Duration: 45 minutes
Materials: *Ida, Always* written by Caron Levis and illustrated by Charles Santoso, crayons, pencil, reflection worksheet
Lesson Objectives: Through the reading of *Ida, Always* students will have an opportunity to process the loss of their classmate. *Ida, Always* is the story of two bears (Ida and Gus), who share a deep friendship. Ida is faced with a terminal illness, and Gus must figure out how to live without his best friend. Through this read-aloud, students will begin to process what life without their classmate will look like. The teacher will help students process their unanswered questions regarding the death of the classmate.

Activating Prior Knowledge: The teacher can begin the lesson by asking students to preview the cover of the book and describe what they see. To prompt discussion, the teacher might ask: "Who do you think these two characters are?," "What do you think their relationship is to one another?," "How do they look when they are together?," and "What do you think this book is going to be about?"
The teacher can provide a brief summary of the book by saying:
"This book is about two friends, Ida and Gus. This is the story of their friendship. As we read this book, I want you to think about ways you might be similar to Gus, one of the bears in the story."

Main Activity: The teacher reads *Ida, Always* to the class. Below are sample questions that may be posed to students:
• "On this page, we learned about some of the activities Gus and Ida liked to do together. We read about how they enjoyed splashing in the water and chasing after one another. What are some of your favorite activities to do with your best friend?"
• "Throughout the story, Gus tells Ida how he would like to see the city. What does Ida say to comfort him?" (you don't have to see it to feel it)
• "What do you think it felt like for Gus to realize Ida would die?"
• "Although Ida is not sure where she will go when she dies, she is sure she will be able to smell Gus' stinky breath. Gus laughs when Ida shares that with him but then wonders if it is okay to laugh. Why do you think this is?
• "Gus and Ida sometimes needed moments alone. Why do you think this is? What do you like to do when you need a moment alone?"
• "Gus and Ida tell each other they miss each other when they part ways. How do you show people you care about them?"
• "How do you think Gus feels when has to do things without Ida by his side?"
• "Throughout the book we hear Ida tell Gus, 'You don't have to see it to feel it.' How does this relate to the death of the deceased classmate?"

• "How does the story end?"

Use the following question to lead into the next activity: "I know many of us are missing ___________, but similar to Gus, we can keep memories of ________ in our hearts. On your desks, there is an activity that allows you to reflect on your favorite images and memories with ___________. In the heart, you may draw your favorite memory with _________. Put them inside your heart, so you can hold them close, always."
The students will be able to take this activity home as a reminder of the ways in which they can maintain bonds with those they love. It will help reinforce the idea that students can hold a piece of _________ in their heart.

Closing: When students finish their illustrations, invite them back to the carpet and provide them with an opportunity to share. This opportunity may be extended by making copies of each student's drawing and binding them into a book for the deceased child's family if the teacher assesses that it would be meaningful to the family.

Additional considerations: We recognize that educators may encounter deaths that are caused by violence, suicide, or natural disasters. The following children's books speak of death more broadly, and thus, can be selected in lieu of *Ida, Always*.
Buscaglia, L. (1982). *The fall of Freddie the leaf: A story of life for all ages.* Slack Incorporated.
Dorn, A. (2022). *When someone dies: A children's mindful how-to guide on grief and loss*. PESI Publishing, Inc.
Karst, P. (2018). *The invisible string* (J. Lew-Vriethoff, Illus.). Little, Brown Books for Young Readers.
Kransy Brown, L. (1998). *When dinosaurs die: A guide to understanding death* (M. Brown, Illus.). Little, Brown Books for Young Reader.
Penn, A. (2009). *Chester raccoon and the acorn full of* memories (B. Gibson, Illus.). Tanglewood.
Rowland, J. (2017). *The memory box: A book about grief* (T. Baker, Illus). Sparkhouse Family.

Name: ____________________
Directions: Think of your favorite memories with _____________.
Draw them inside your heart, so you can hold them close, always.

I keep ___________________ with me,
