# Supplementary material for: Helping Children Cope with Loss: Legacy Interventions for the Grieving Classroom
Source: Contin Educ. 2022 Sep 5;3(1):92–100. doi: 10.5334/cie.45 (PMC11104402; doi:10.5334/cie.45)
Supplement: Appendix B. — Middle school and high school legacy building sample lesson plan. [file cie-3-1-45-s2.pdf]

Appendix B

Middle School and High School Legacy Building Sample Lesson Plan

Plan: Memories That Bind
Subject: English Language Arts, Writing, or Art
Grades: 6 -12
Duration: One hour
Materials: One string art kit for each student, consisting of a square of foam, cork, or wood with pre-hammered, raised nails or carpet tacks in a shape or pattern (may need preassembly for use); embroidery floss in varied colors, paper, writing utensils, access to an audio speaker, and a digital device with photography capabilities. For a more time-limited or less physically complicated version, two popsicle-style craft sticks and yarn may be substituted.
Objectives: Through a guided listening and breathing exercise, reflective dialogue, and participation in an open-ended art activity, students will explore the concept of grief as well as strategies for expressing and coping with their own experience of grief.
Notes for the teacher: Depending on your students' interests and comfort with different media modalities, you may want to choose a different piece of music or poetry/prose to open the activity and invite space for reflection.

Activating Prior Knowledge: Students will likely need several sensory cues to help them shift their focus to a more personally focused and reflective environment. This can be done through a guided listening exercise, which the teacher can choose to introduce by saying something like:
"As you all may know and feel, we are missing ____ in our classroom today because they have died. Death is a really hard thing to make sense of, no matter how old or smart you are, and it can bring big and complicated feelings that are hard to sort through. Those feelings are different for everyone and can change from minute to minute. Perhaps you have been feeling sad in your grief, or frustrated, or confused, or perhaps you have had a hard time feeling anything. Grief takes many forms, and there is no right or wrong way to feel and grieve. Today I'd like to try something, for you and for me, in our grief, to help us recognize how we are feeling and remember our friend ______."
The teacher can then give each student a blank piece of paper and writing utensil (as needed), explaining, "We are going to do a listening exercise for a few moments. I am going to play/read a song/poem/story for us, and I'd like you to feel free to use your paper and pen to doodle, write, or just be still as you hear the sounds/words and think about _________."
Suggested Media (these selections may be substituted and adapted to reflect the cultural background of the students):
• Songs (with words)
o "Keep Me in Your Heart" by Warren Zevon
o "Remember Me" by Miguel, from Disney's movie *Coco*
o "Fire and Rain" by James Taylor

• Songs (instrumental)

o “San Solomon (Reprise” by Balmorhea
o “I Remember” by GAEL
o “For Those We Lost” by Oskar Kappland

• Poetry/Prose
o “Blessing for the Brokenhearted” by Jan Richardson
o “Sorry for Your Troubles” by Padraig Tuama
o “How Night Came Into Being” Hindu folktale
o “On the Death of the Beloved” by John O’Donohue

Main Activity: The teacher begins playing the chosen song or reading the selected/poetry prose as students listen. Once the piece of music or reading is complete, the following questions can be used to invite dialogue; teachers may assess students as more apt to respond if they are given time to reflect in small groups and can choose to make this adjustment.
• “Tell me about some of the images, phrases, or feelings that came into your mind as you were listening.”
• “What were some of the feelings, sounds, or words that felt important to you?”
• “What do you think the composers/poets/writers were envisioning when they wrote this piece?”
• “How were the composers/poets/writers feeling when they wrote this piece?”
• “What would __________ think or have to say about the song/poem/story we just heard?”
The teacher can use a statement such as the following to lead into the next portion of the activity:
“Grief is a combination of feelings that we experience after someone has died or after we experience a loss. For centuries, music, art, and literature have been ways that different cultures have used to describe or express their feelings of grief. Sometimes their sounds and words were focused on the sadness or anger that they felt, while other times the sounds and words represented the person or loved one that was meant to be remembered – their legacy.
_______ left a legacy in our classroom by the words they said, the things they did, and the relationships they built with us. To honor _________’s legacy, I’ve put together an art project for you to work on independently. You’ll see that there are a variety of strings up front to symbolize how we are each connected to the people we love – friends, family, pets, ___________. As you are choosing which colors to weave around the poles/popsicle sticks, think about how those colors make you feel, who they remind you of, or what memories they are connected to. As you weave around the poles/popsicle sticks, think about how these feelings, memories, people, and ________ are also connected, and how they have helped to make you who you are today. Notice how the threads and colors wind over, under, and around one another. Notice how hard it is to tell where one string begins and another ends. So too are the legacies of those we have come to know and love, pieces of ourselves that are hard to untangle, but help to make us who we are.”
As students finish their art pieces, the teacher can invite them to talk about them with the class if they would like. If students do not want to share, the teacher can share

their art piece and discuss the reasons they chose each color and the design they intended to create as an example. The teacher should again validate the normalcy of grief, the variety of emotions that grief can evoke, and the value of memories for staying connected to those that have died.

Concluding: The teacher can ask students to turn over their art piece and, using a writing utensil or permanent marker, write two things they can do to cope with grief. The teacher might say, “Write down two things that you can do or use to help you when your grief is feeling especially hard.” The teacher can then invite discussion about what students chose to write, emphasizing that coping looks different for every person, and the importance of letting others cope in the way that works for them.

To close the activity, the teacher can invite students to have a photo of their art taken and added into a photo album for the deceased student’s family. The teacher should also use this opportunity to remind students of the counseling services available at the school and in the community and encourage them to talk with a trusted adult if they have lingering questions or concerns.
